# Pillararenes as Promising Carriers for Drug Delivery

**DOI:** 10.3390/ijms24065167

**Published:** 2023-03-08

**Authors:** Grigory V. Zyryanov, Dmitry S. Kopchuk, Igor S. Kovalev, Sougata Santra, Adinath Majee, Brindaban C. Ranu

**Affiliations:** 1 Chemical Engineering Institute, Ural Federal University, 19 Mira Street, 620002 Yekaterinburg, Russia; 2 I. Ya. Postovskiy Institute of Organic Synthesis, Ural Division of the Russian Academy of Sciences, 22 S. Kovalevskoy Street, 620219 Yekaterinburg, Russia; 3 Department of Chemistry, Visva-Bharati University, Santiniketan 731235, India; 4 School of Chemical Sciences, Indian Association for the Cultivation of Science, Jadavpur, Kolkata 700032, India

**Keywords:** pillararenes, host–guest complexes, aggregates, antibiotics, cytotoxic drugs, insulin, drug loading, drug release

## Abstract

Since their discovery in 2008 by N. Ogoshi and co-authors, pillararenes (PAs) have become popular hosts for molecular recognition and supramolecular chemistry, as well as other practical applications. The most useful property of these fascinating macrocycles is their ability to accommodate reversibly guest molecules of various kinds, including drugs or drug-like molecules, in their highly ordered rigid cavity. The last two features of pillararenes are widely used in various pillararene-based molecular devices and machines, stimuli-responsive supramolecular/host–guest systems, porous/nonporous materials, organic–inorganic hybrid systems, catalysis, and, finally, drug delivery systems. In this review, the most representative and important results on using pillararenes for drug delivery systems for the last decade are presented.

## 1. Introduction

Pillararenes (PAs), new macrocyclic hosts, were introduced for the first time as pillar[5]arenes (P5As) by Ogoshi and co-workers in 2008 [1]. In 2009, pillar[6]arenes (P6As) were reported for the first time by Meier and co-authors [2]. Since then, many other synthetic approaches to P5-6As were reported [3,4,5], and, now, up to pillar[15]arenes have been reported [6]. Due to their rigid penta- or hexagonal shape with hydrophobic and high-electron rich cavities of 5.5–7.5 Å in diameter [1,7], P5–6As have already found wide paratactical applications. Thus, PAs demonstrate their excellent capacity to encapsulate a large variety of cationic guests, including ferrocenyl cations [8], as well as a large variety of neutral guests [9], amino acids [10], including the recently reported example of tyrosine encapsulation to inhibit tyrosine phosphorylation in cells [11]. These supramolecular properties of PAs can be modulated by means of the proper functionalization of both ends of the macrocyclic cavity in order to provide, for instance, better water solubility [12,13,14,15,16,17,18,19,20] or amphiphilic character [21]. Thanks to all above mentioned PAs are heavily involved in many research fields and applications, such as stimuli-responsive supramolecular/host–guest systems [8,9,10,11], porous materials [22,23,24,25], as materials for gas storge/separation [26,27,28,29,30], organic–inorganic functional materials [19,31,32], (photo)catalysts [33,34,35,36], molecular devices and machines [37], supramolecular polymers with host–guest interaction that enable conductivity [38], fluorescent and aggregation-induced emission (AIE) materials [39,40], materials for DNA fragments extraction [41], and nanotheranostics [42,43,44,45].

The application of PAs as carriers for the targeted drug delivery systems is of particular interest [46,47,48], as, depending on the structure of PA-based carriers, disease type, and the nature of the drug, various factors for the drug activation/release can be utilized, such as drug release upon the chemical reaction with intercellular compounds, for instance, thiols, amines, reactive oxygen species (ROS), etc., drug release in acidic *pH* media, hypoxia, certain types of enzymes, and light-activated release. Based on all the above, PA-based drug delivery systems are arranged in this present review according to a disease type and drug activation/release mechanism.

## 2. Pillararene-Based Antibacterial Systems

The high drug resistance of pathogenic bacteria is, in most cases, accompanied by biofilm formation, which makes bacteria tolerate antibiotics, host defense systems, and other external stresses, and, thus, it contributes much to persisting chronic infections [49,50,51]. Within such biofilms, bacteria are protected from desiccation, immune system antimicrobials attack, ingestion by protozoa, antibiotics, etc. [52], and most microbial cells demonstrated 10–1000 times more antibiotics resistance compared to the cells in a planktonic state [53,54,55,56]. According to the literature [57], depending on the bacteria type and the nature of an antibiotic drug, different mechanisms will account for antibiotic resistance, while the environmental heterogeneity within the biofilm might promote the formation of a heterogeneous population of cells with different levels of resistance. In this regard, PAs can serve both as transport systems to help antibiotics to penetrate the biofilm better and as compounds preventing/inhibiting biofilm formation.

Regarding the last one, Cohen et al. reported water-soluble cationic PA5s and PA6s bearing different positively charged quaternary ammonium or imidazolium groups to efficiently inhibit the formation of biofilms by Gram-positive pathogens, such as *S. aureus subsp. aureus Rosenbach ATCC 33592*, *S. aureus ATCC 29213*, *S. aureus BAA/043*, *E. faecalis ATCC 29212*, *S. epidermidis RP62A*, and *S. mutans ATCC 700610* [58], with a minimum biofilm inhibitory concentration (MBIC50) value from 0.4 to 6.4 μM. For the PA5s bearing more hydrophilic hydrophilicity hydroxyethyl-dimethyl quaternary ammonium groups, no inhibition of biofilm formation was observed at the highest concentration tested, which was 12 μM. None of the cationic PAs inhibited the formation of biofilms by Gram-negative strains, such as *E. coli ATCC 25922* and *P. aeruginosa PAO1*. No antibiofilm activity was found for tetramethylammonium bromide (TMA-Br) or tetramethylammonium chloride (TMA-Cl) in concentrations up to 208 and 292 μM, respectively, indicating that neither halogen ions nor the quaternary ammonium head groups are responsible for the antibiofilm activity. In addition, for the negatively charged groups-appended pillar[5]arene, no antibiofilm activity was observed.

Simultaneously, the same group investigated symmetric PA5s and their monomeric analogues bearing ethyl- and methyl-phosphonium units [59]. The tests against methicillin-resistant *S. aureus ATCC 33592* and *E. faecalis ATCC 29212* demonstrated excellent biofilm-inhibiting activity with MBIC50 values of 0.17 to 0.47 μg mL^−1^ for both bacteria, while monomeric analogues did not show any activity.

Finally, the same group evaluated the biofilm formation-inhibiting activity of 16 cationic pillar[5]arene derivatives, including the cationic water-soluble pillar[5]arene-based rotaxane [60]. According to the authors, positively charged head groups are critical for the observed antibiofilm activity. The authors have found that a plurality of accessible positive charges are important determinants for the observed antibiofilm activity. It was found that the multiplication of the number of positive charges from 10 to 20 did not increase the antibiofilm activity. The authors also suggested that the lipophilicity is also important, as pillararenes with five positive charges and five long alkyl chains exhibited a reduced antibiofilm activity. The cavity of the pillar[n]arene was found to be not so critical for the above-mentioned activity, although the pillar[n]arene core appears to be important, as it facilitates the clustering of the positive charges. In addition, pillararenes were nonhemolytic at concentrations that were ∼100-fold of their MBIC_50_. Based on all the above, the authors suggested that PAs can serve as hosts for small molecules and that aliphatic chains are not critical for the antibiofilm activity. However, the clustering of the positive charges on the PA skeleton is important. Finally, the authors mentioned that PAs selected as potent inhibitors of biofilm formation were nonhemolytic at concentrations that are ∼100-fold of their MBIC50 and did not have an effect on bacterial cell growth.

Another approach to inhibit biofilm formation is based on sugar–lectin interactions. In this regard, several PAs bearing a sugar moiety were reported. Thus, Imberty et al. described asymmetric pillar[5]arenes bearing galactose and fucose moieties [61]. According to the authors, the galactosylated PAs bound to LecA (*Pseudomonas aeruginosa*) and the fucosylated derivatives bound to the fucose-specific lectins LecB (*Pseudomonas aeruginosa*) and BambL (from *Burkholderia ambifaria*). This specificity would undoubtedly impart biofilm inhibition where *BambL*-expressing bacteria were involved. Nierengarten et al., by using azide–alkyne “click” conjugations, prepared PA5s bearing up to 20 peripheral sugars from one or both sides of the PA core [62]. It was said that the galactosylated PAs with a longer substituent bound to LecA more efficiently, and binding of the fucosylated PAs to LecB also increased with linker length. PAs with 20 fucose groups were also superior to their analogues bearing 10 units. Here, the main challenge is the stereochemistry of the PA5 core, as the obtained compounds were obtained as mixtures of diastereoisomers. Simultaneously, the same group prepared a number of rotaxanes based on PA5s [63] by using a similar synthetic approach. As a result, fucose- or galactose-substituted PA5s were obtained, and such a combination of galactose (10 units) and an additional 2 fucose units within the PA moiety was highly promising for the possible applications of the obtained supramolecular assembly for the targeting of 2 bacterial lectins (LecA and LecB) from the opportunistic pathogen *Pseudomonas aeruginosa*.

Several PA-based antibiotic drug transport systems were described. Thus, Li et al. reported epoxy-substituted P5As (PAEP) possessing a unique, pillar-shaped, three-dimensional macrocyclic structure [64]. The authors claimed that the PAEP exhibited a strong host–guest complexation with 1,4-dibromobutane (DBrBu), a potential organic antibacterial agent, with a complex constant of 215.7 ± 11.02 M^−1^.

The above-mentioned negatively charged P5As did not show any antibiofilm activity [58]. However, due to the acidic microenvironment of an infection caused by, for instance, lactic acid accumulation [65], the negatively charged P5As might be used as targeted delivery systems for antibiotic drugs. Keeping this in mind Pisagatti, Notti, and co-authors described deca-carboxylato-substituted P5As as a carrier for the aminoglycoside antibiotic amikacin [66]. Under physiological conditions, this anionic P5A demonstrated a quite efficient binding of amikacin (Kass = 9.9 ± 1.28 × 10^3^ M^−1^), thus allowing the modulation of the in vitro antimicrobial activity of this drug towards *S. aureus*.

**Figure 1:**
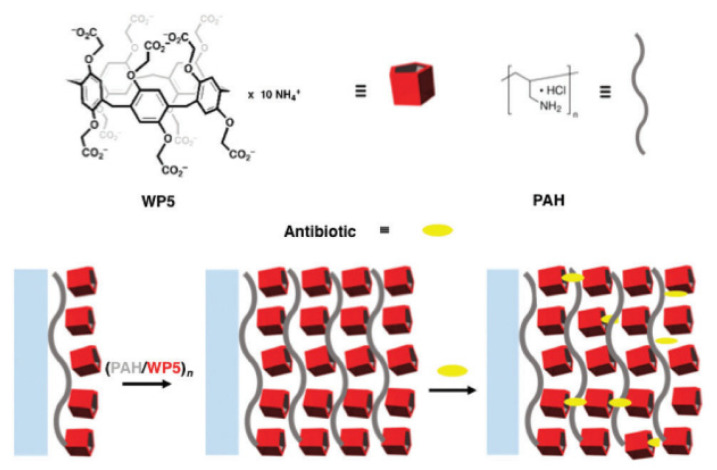
The formation of “**WP5**–antibiotic” inclusion complexes. Reproduced with the permission of reference [67]. Copyright © Royal Society of Chemistry 2018.

Later, the same group reported their results on constructing carboxylato-pillar[5]arene (WP5)/poly(allylamine hydrochloride) (PAH) films for the sustained release of antibiotics, such as levofloxacin and amikacin [67] (Figure 1). The formation of a “WP5*levofloxacin” inclusion complex was monitored in the nuclear magnetic resonance (NMR) timescale, and the piperazine-ring resonances of the guest underwent substantial up-field shifts of the signals of protons of WP5/PAH moieties as a consequence of the magnetic shielding of the host cavity. In addition, the 2D NOESY spectrum showed intermolecular correlation peaks between the ArH and OCH_2_ moieties of WP5 and several hydrogen atoms of levofloxacin. In addition, films loaded with levofloxacin or amikacin were found to be extremely efficient in inhibiting bacterial colorizations, causing, after 2 h, a dramatic decrease of steady and persistent colony-forming units (CFU) (10^2^–10^3^-fold). In in vitro experiments with Gram-negative and Gram-positive bacterial strains (i.e., *Pseudomonas aeruginosa ATCC27853* and *Staphylococcus aureus ATCC29213*), the quantities of levofloxacin released from the multilayer (2 and 3 mg mL^−1^ after 5 and 480 min, respectively) were found to closely match the MIC_90_ values specific to the levofloxacin/*P. aeruginosa* and levofloxacin/*S. aureus* pairs (2 and 1 mg·mL^−1^, respectively).

**Figure 2:**
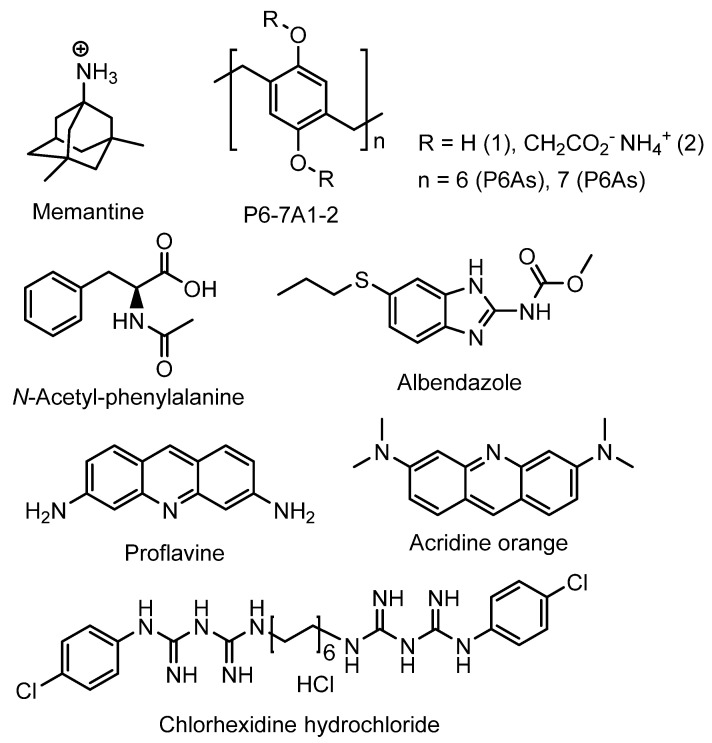
**P6A**- and **P7A**-based hosts and small molecules-based guests.

Wheat and co-authors described carboxylated P6As and P7As as hosts for various guests, such as memantine, chlorhexidine hydrochloride, and proflavine, to study the possible application of these PAs for drug delivery and biodiagnostic applications [68] (Figure 2).

The authors claimed that both PAs were able to form host–guest complexes with small guest molecules/drugs, and this was confirmed by means of ^1^H NMR and theoretical modeling experiments. The encapsulation is promoted by the suitable cavity size along with the combinations of hydrophobic effects, hydrogen bonding, and electrostatic interactions at the portals. Based on the theoretical calculations and docking experiment, proflavine and **P7A2** form a 2:1 host–guest complex with a binding affinity of −10.1 kcal/mol, while, with other guests, both PAs formed 1:1 inclusion complexes with binding affinity values varying from −2.9 to −8.9 kcal/mol. In addition, for the fluorescent proflavine, its encapsulation by **P6A2** resulted in dramatic fluorescence quenching (a static quenching mechanism was suggested), and the observed fluorescence on/off were usable for biodiagnostics purposes. In addition, the toxicity of P6As and P7As was examined using in vitro growth assays with the human embryonic kidney (HEK) cell line HEK293 and the human ovarian carcinoma cell line OVCAR3. According to the authors, both PAs are relatively nontoxic to the cells, although they inhibit proliferation at high doses (500 mM) and at extended time periods (over 78 h). Based on all information mentioned above, **P6-7A1-2** may have an application in drug delivery.

**Figure 3:**
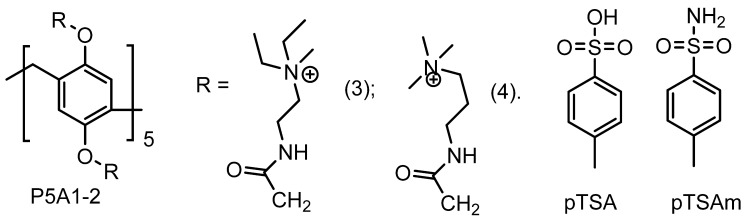
**P5A1-2** as hosts for pTSA and, possibly, for pTSAm.

Stoikov et al. reported synthesis of water-soluble cationic pillar[5]arenes **P5A1-2** and studies of their inclusion complexes with *p*-toluenesulfonic acid (pTSA) [69] (Figure 3). The formation of a 1:1 “P5A*pTSA” inclusion complex was confirmed based on the data of 1D ^1^H and 2D NMR spectroscopy and UV spectroscopy. The *log*K*_ass_* values of the complexes **P5A1** and **P5A2** with pTSA were 1.22 ± 0.08 and 1.43 ± 0.12, respectively. Owing to the ability of cationic PAs to inhibit biofilm formation reported by the authors, P5As may be used as possible carriers for the transport of the simplest antibiotics derived from pTSA, such as sulfanilamide (pTSAm) [70].

**Figure 4:**
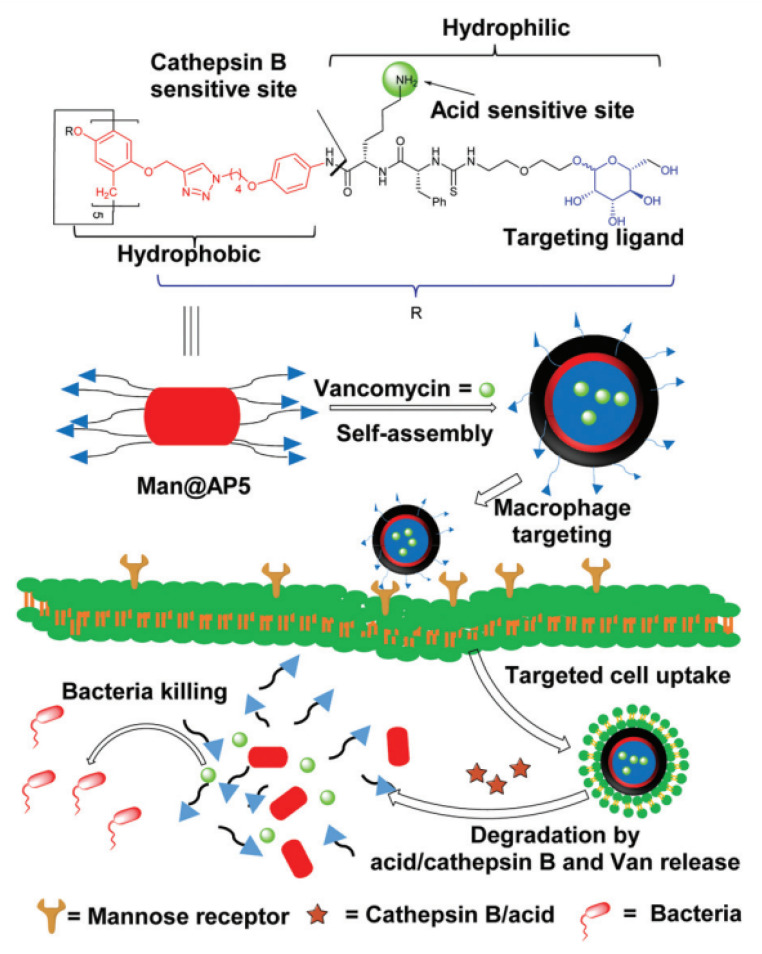
Schematic illustration of a Van-loaded mannosylated vesicle (Man@AP5-Van) and its targeted uptake, transport, degradation, Van release, and bacterial inhibition. Reproduced with the permission of reference [71]. Copyright © Royal Society of Chemistry 2020.

It is known that some bacteria such as Methicillin-resistant *S. aureus* (MRSA), by means of invading and colonizing inside phagocytes such as macrophages, can develop self-protection mechanisms to protect themselves against antibiotics, as well as to escape from host phagocytosis. In attempts to discover advanced therapeutics for bacterial infections and cancers, Xie, He, and co-authors reported self-assembled architectures of amphiphilic P5As (**Man@AP5**) with encapsulated vancomycin (“Man@AP5*Van”) [71] (Figure 4).

To target macrophages, which express high levels of mannose receptors [72], mannose moieties were introduced into P5As. **Man@AP5** self-assembles to form a vesicle that encapsulates Van (“Man@AP5*Van”). The authors claimed that, inside the macrophages, “Man@AP5*Van” protonation takes place, and the Phe-Lys linker is cleaved by cathepsin B in the lysosome [73]. The photolysis of the “Man@AP5*Van” vesicle results in its degradation and release of Van to effectively inhibit intracellular MRSA growth (Figure 2). According to the authors, the main advantages of **Man@AP5** vesicles for Van delivery are (1) macrophage targeting for antibiotic accumulation with reduced side effects; (2) *pH* and cathepsin B dual responsivity for rapid drug release; (3) good biocompatibility and biodegradability. Van was selected by the authors owing to its high efficiency for the treatment of bacterial infections. Due to poor intracellular antibacterial activity [74] and low uptake by infected host cells, Van could be used to treat intracellular staphylococcal infections only at a high concentration (100 mg·mL^−1^), and it exhibited no significant activity against intracellular MRSA at low concentrations. Along with the mannose-substituted P5As “Man@AP5*Van”, for the Van loading efficiency, PEG-substituted P5A-based vesicles were used (“mPEG@AP5*Van”), and the drug-loading efficiency of Van-loaded **Man@AP5** (“Man@AP5*Van”) or Van-loaded “**mPEG@AP5**” (“mPEG@AP5*Van”) was 41.6% or 34.1% with the drug loading content of 25% or 25.4%, respectively. The encapsulation of Van by the **Man@AP5** and **mPEG@AP5** vesicles was confirmed by the data of transmission electron microscopes (TEM) (“Man@AP5*Van” and “mPEG@AP5*Van” vesicles showed darker interiors and were larger than the blank vesicles) and by dynamic light scattering (DLS) experiments. The efficient extracellular release of Van from “Man@AP5*Van” and “mPEG@AP5*Van” vesicles in acidic media (99% for both at *pH* 5.0 for 5 h, and *pH* 6.0 for 7 h) and upon the exposure to different concentrations of cathepsin B (the amount of free Van from “Man@AP5*Van” reached a maximum of 99% with 2.5 mg·mL^−1^ of cathepsin B after 50 min, and 1.5 mg·mL^−1^ of cathepsin B after 80 min) or, at the faster rate, upon the combined treatment, was observed. These results suggested the high sensitivity of “Man@AP5*Van” vesicles to cathepsin B cleavage for rapid Van release. According to the results of studies of extracellular antibacterial activity of Van-loaded vesicles, “Man@AP5*Van” and “mPEG@AP5*Van” did not inhibit the growth of MRSA WHO-2 at the same Van concentration (5 mg·mL^−1^), while, in the presence of cathepsin B (1.5 mg·mL^−1^) or at the *pH* of 5.0, “Man@AP5*Van” and “mPEG@AP5*Van” exhibited comparable bacterial inhibition to free Van (5 mg·mL^−1^) after 24 h, supporting that Van release could be triggered by the acidic *pH* or cathepsin B. Finally, the ability of “Man@AP5*Van” and “mPEG@AP5*Van” to inhibit intracellular bacteria was studied by counting the CFU of surviving intracellular bacteria after incubating these vesicles with MRSA WHO-2 strain-infected Raw264.7 cells. “Man@AP5*Van” exhibited a significantly stronger inhibition compared to “**mPEG@AP5-Van**” or free Van, and this could be accounted for by its efficient Van delivery to and Van release in bacterial-infected Raw264.7 cells. In addition, upon the incubation of MRSA-infected macrophages with free Van, “Man@AP5*Van”, or “mPEG@AP5*Van” at 10 mg mL^−1^, the “Man@AP5*Van” exhibited better intracellular inhibitory activity compared to “mPEG@AP5*Van” or free Van. Based on all the above, the authors conclude that the “Man@AP5*Van” was the best delivery system for the Van inside the RAW264.7 cells and, thus, exhibited significantly stronger inhibitory effects against intracellular MRSA. It is worth mentioning that both empty vesicles and Van-loaded vesicles have no cytotoxic effect against 293T, HUVEC, and RAW264.7 cells.

Yang, He, and co-authors reported P5A-based nanoparticles for targeted antibiotic delivery for treating methicillin-resistant *S. aureus* [75]. To do that, mannose-modified pillar[5]arene was assembled for the preparation of a glutathione (GSH)-responsive multifunctional antibiotic delivery system (“WP5A*G”) based on the host–guest interaction between P5A and a near-infrared (NIR) fluorescence receptor (**G**), which was successfully developed. “WP5A*G” could encapsulate hydrophobic antibiotics such as linezolid (LZD) to form drug-loaded nanoparticles (“LZD*WP5A*G”), improving the anti-intracellular MRSA activity of LZD with excellent biocompatibility. This easily prepared and flexible system could be employed for simultaneous non-invasive cellular imaging and real-time monitoring of antibiotic release, representing a novel strategy toward targeted antibiotic delivery into macrophages for treating intracellular bacterial infections.

The “WP5A*LZD” vesicles exhibited GSH-triggered LZD release, with up to 90% release in 40 min at 3 mM concentration of GSH.

## 3. Pillararene-Based Systems for the Targeted Chemotherapy of Cancer

For various cancers, the tumor microenvironment is characterized by hypoxia-driven higher levels of ROS, such as hypochlorous acid (HOCl), hydrogen peroxide (H_2_O_2_), hydroxyl radicals (OH•), and singlet oxygen (^1^O_2_) [76,77], higher *pH* (7.4) [76] or lower *pH* (*pH* 5.5–6.0 in endosomes and *pH* 4.5–5.0 in lysosomes of tumor cells) [78], and, finally, overexpressed endogenous thiols, including glutathione (GSH), thioredoxin (Trx), cysteine (Cys), hydrogen sulfide (H_2_S) with 2–3 orders of magnitude higher than the levels in the extracellular environments [76,79], polyamines [80], adenosine triphosphate (ATP) [81], etc. Therefore, by means of proper synthetic design, one can construct PA-based transport systems, which are able to properly encapsulate drug or prodrug cytotoxic molecules and release them selectively inside the tumor microenvironment. Below, the various PA-based anticancer drug carriers are analyzed and arranged based on the stimuli for drug release.

## 4. (Bio)thiol-Triggered Pillararene Drug Delivery Systems

**Figure 5:**
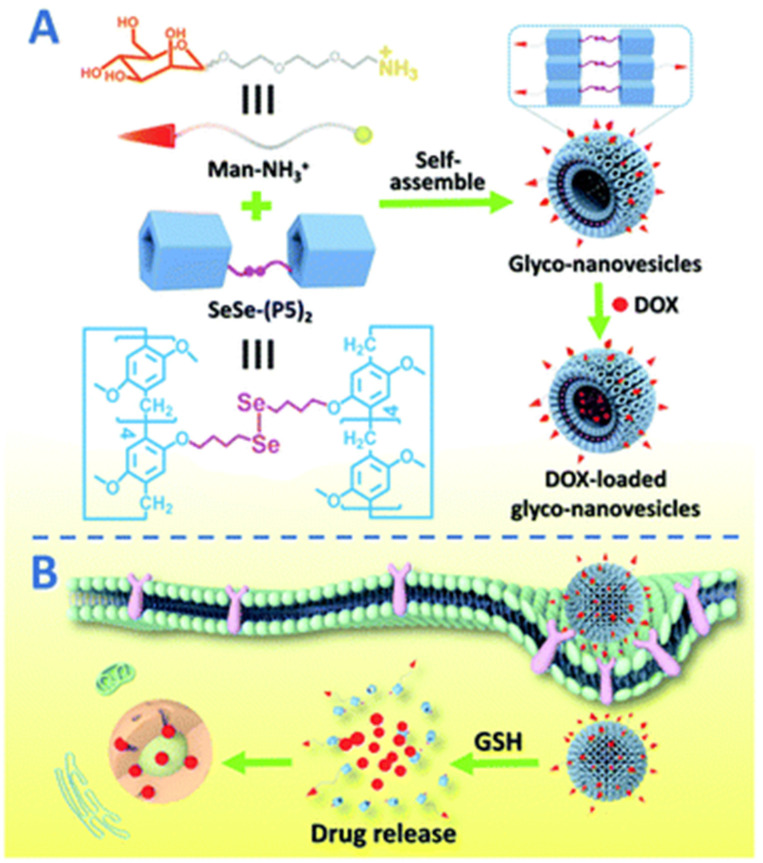
Schematic representation of (**A**) the construction of supramolecular glyco-nanovesicles “(SeSe-(P5)2*Man-NH_3_” and (**B**) their targeting chemotherapy. Reproduced with the permission of reference [82]. Copyright © Royal Society of Chemistry 2020.

Pei and co-authors reported a nano-drug delivery system based on the supramolecular assembly of P5A-based Se-Se-linked dimeric molecules (**SeSe-(P5)_2_**) with encapsulated mannose-based guests (**Man-NH_3_^+^**) [82,83] (Figure 5).

In H_2_O/THF (10:1) solution, “SeSe-(P5)_2_*Man-NH_3_^+^^”^ forms glyco-nanovesicles, which was confirmed by SEM and TEM, with hollow spherical morphology. Upon incubation with HepG2 human hepatoma cells, MCF-7 breast cancer cells, and 293T healthy human cells, even in high concentrations (200 mg × mL^−1^), “SeSe-(P5)_2_*Man-NH_3_^+^” glyco-nanovesicles did not show any cytotoxic activity. After the loading of doxorubicin hydrochloride (DOX), the glycol-nanovesicles changed their morphology to irregular nanoparticles, which was confirmed by SEM. After being incubated with GSH (10 mM) under neutral conditions, the DOX incapsulated into glycol-nanovesicles was inappreciably released with the cumulative release amount of 13% within 72 h, and a 2.5 mM GSH cumulative release amount of 62% was detected. This confirms the ability of PA5-based glyco-nanovesicles for GSH-responsive DOX release in tumor microenvironments. In addition, the in vitro anticancer efficiency of the DOX-loaded glyconanovesicles was investigated with respect to HepG2 and MCF-7, and, based on the data of MTT tests, these “SeSe-(P5)_2_*Man-NH_3_^+^” glyco-nanovesicles demonstrated good targeting ability and cytotoxic effect against cancer cells, along with reducing the toxicity to normal cells.

**Figure 6:**
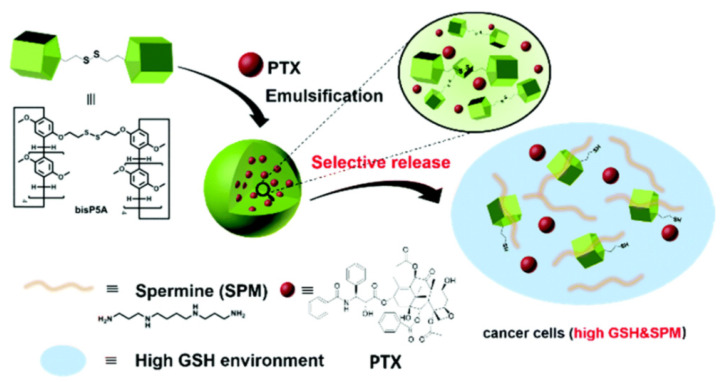
Schematic illustration of the formation of bisP5A NPs and their stimuli-responsive drug release. Reproduced with the permission of reference [84]. Copyright © Royal Society of Chemistry 2019.

Wang and co-authors reported a similar approach and obtained P5A-containing nanoparticles by using disulphide-bridged P5A and poly(vinyl alcohol) (**bis-P5A**) [84] (Figure 6). The obtained NPs were loaded with paclitaxel to form paclitaxel-loaded NPs, and the drug encapsulation efficiency (DEE) and drug loading efficiency (DLE) of PTX-NPs were determined to be 84.5% and 11.5%, respectively. In the presence and in the absence of biothiol, such as GSH (10 mM), PTX-NPs exhibited excellent stability, with accumulated PTX release of less than 17% after incubation for 86 h. Similarly, when incubated with a polyamine, such as spermine (SPM), which is overexpressed in several types of cancer cells (e.g., prostate cancer, lung cancer, and breast cancer), taken at the concentration of 1 mM, the PTX-NPs exhibited excellent stability, with less than 11% of accumulative drug release. Finally, in the presence of both 10 mM GSH and 1 mM SPM (both are present inside cancer cells such as lung cancer cells) the authors observed a cumulative drug release of up to 65% after incubation for 86 h. In the presence of GSH (1 mM) and SPM (0.1 mM), which are simulated concentrations in non-cancerous cells, the PTX-NPs demonstrated PTX release of 27%, which is relatively slow. In addition, more rapidly, PTX was released in the presence of both 10 mM GSH and 1 mM SPM, with the cumulative drug release amount of up to 65% after 86 h incubation. Finally, models for in vitro experiments overexpressing GSH and SPM cancer cells were taken, such as A549 human lung cancer cells, as well as L02 healthy human liver cells. According to the authors, L02 cells exhibited comparable cellular viabilities with respect to both PTX and PTX-NPs with the IC_50_ (50% of cell growth inhibition concentration) values of 16.95 nM (PTX) and 16.47 nM (PTX-NPs), respectively, while in case of GSH- and SPM-overexpressed A549 cells, IC_50_ values for free PTX (22.23 nM) were about 10 times higher compared to PTX-NPs (2.76 nM), which is probably due to the precisely selective release of PTX inside these cancer cells, and that results in significantly improved cell death via the apoptosis mechanism.

**Figure 7:**
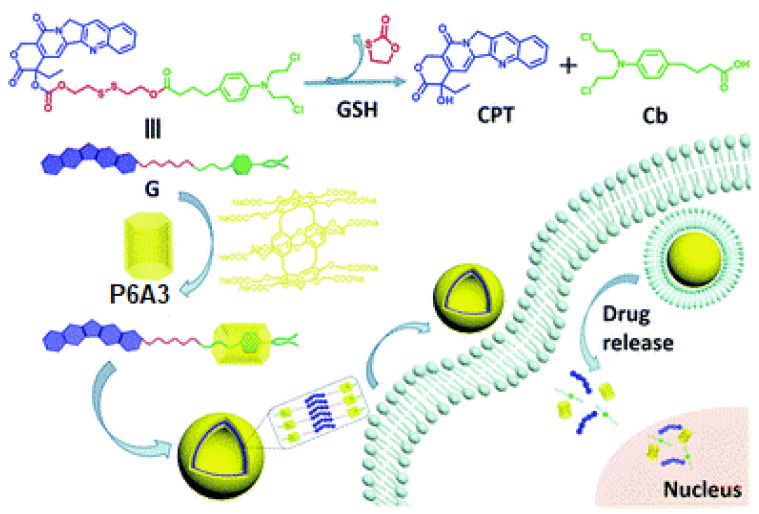
Schematic design of a **P6A3**-supported dual prodrug guest (**G**) system. Reproduced with the permission of reference [85]. Copyright © Royal Society of Chemistry 2018.

The same group reported a P6A-supported dual prodrug guest (**G**) system, in which two different anticancer drugs, camptothecin (CPT) and chlorambucil (Cb), were connected via disulfide linker and assembled with a water-soluble PA6-based host to form a host–guest complex in a ratio of 1:10 with the drug-loading content of 63.8% [85] (Figure 7). The host–guest interactions occurred between **P6A3** and the Cb moiety of the guest, and were confirmed based on 1D ^1^H NMR and 2D NOESY experiments. According to the DLS, **P6A3** and **G** aggregate to form nanovesicles with an average hydrodynamic diameter of 143 nm with a narrow size distribution, while, based on atomic force microscopy (AFM) and TEM data, “P6A3*G” aggregates possess hollow spherical morphology with diameters ranging from 70 nm to 120 nm. It was said that, at the GSH concentration of 10 mM, the disulfide linker cleavage occurs with the drug release up to 95.1% over a period of 72 h at *pH* 7.4. In addition, no vesicle structure could be observed from the TEM image. In addition, based on in vitro studies, it was shown that this dual-drug-loaded system has a comparable antitumor activity toward MCF-7 cancer cells as a 1:1 mixture of CPT and Cb.

**Figure 8:**
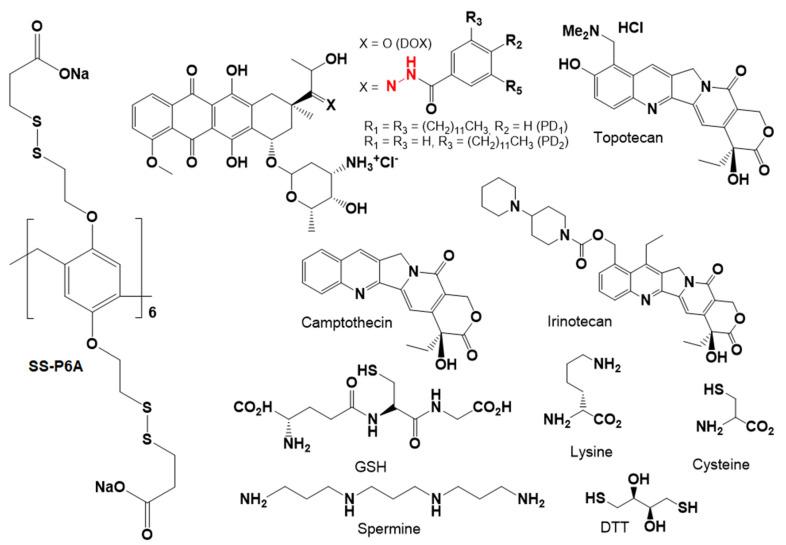
Chemical structures of host **SS-P6A**, anti-tumor drugs and prodrugs, thiols, and spermine.

**Figure 9:**
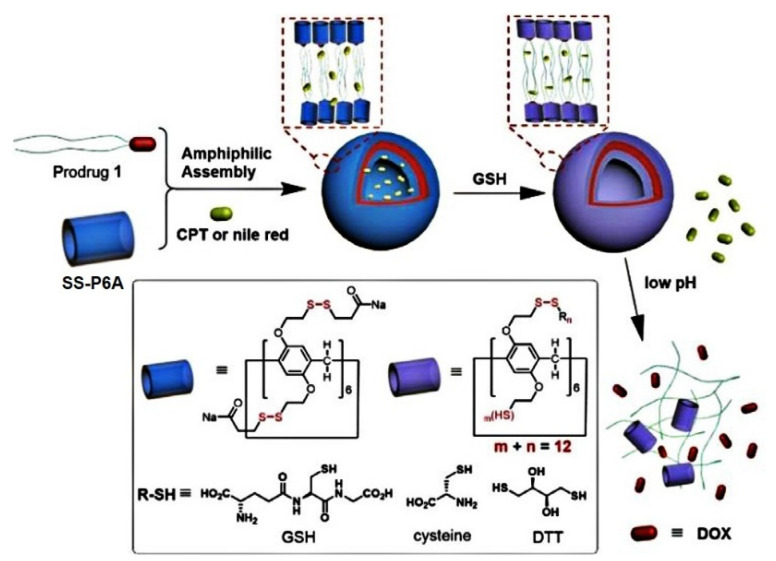
Schematic illustration for the formation of supramolecular vesicles and GSH/acid-induced sequential cargo release. Reproduced with the permission of reference [86]. Copyright © Elsevier 2022.

Ma et al. described a glutathione-responsive drug delivery system based on water-soluble PA6-based substituted 12 disulfide-containing moieties bearing carboxylic acid terminal groups (**SS-P6A**) for improving aqueous solubility and enhancing binding affinity with cationic guests, such as anticancer drugs, amines, and thiols [86] (Figure 8 and Figure 9).

In phosphate-buffered saline, these **SS-P6A** exhibited high binding affinity to DOX, irinotecan, and spermine with up to 10^5^ M^−1^ association constants. For the (bio) thiols (DTT, cysteine and GSH) and amino acid lysine, two orders of magnitude lower binding constants were observed. In addition to DOX, guest molecule **SS-PA6** was able to incapsulate two DOX prodrugs, PD1 and PD2, bearing acid-labile hydrazone linkers, and one or two hydrophobic alkyl chains, and, according to TEM and DLS, vesicle architectures with an average vesicle size for “SS-P6A*PD1” of 49 nm and 135 nm for “SS-P6A*PD2” with a wall thickness of 12.1 nm and 9.3 nm, respectively, were obtained. In addition to DOX-based molecules, hydrophobic drug camptothecin (CPT) or Nile Red dye were encapsulated by **SS-P6A** to form supramolecular vesicles, and, in the presence of GSH, an efficient guest release was observed. For the DOX-prodrugs-loaded **SS-PA6**, the authors observed a *pH*-triggered (*pH* = 4.0) DOX release, while negligible DOX release was observed at neutral *pH*. Finally, in vitro studies using HeLa cancer cells and normal Chang liver cells were carried out, and the ability of CPT-loaded **SS-P6A** for the GSH-mediated release of CPT in HeLa cancer cells and for DOX-loaded **SS-P6A** to release DOX in lysosomes via endosome/lysosome-mediated endocytosis was confirmed.

## 5. Hypoxia-Driven Drug Release Pillararene-Based Systems

**Figure 10:**
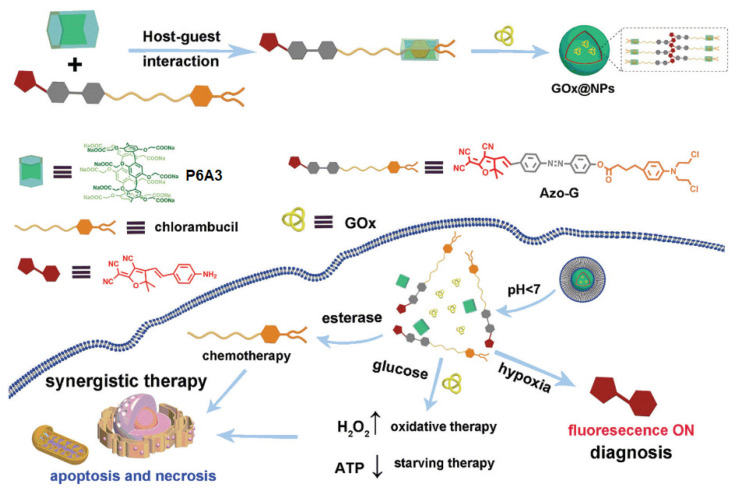
Fabrication of the hypoxia-activatable **P6A3**-based drug delivery system. Reproduced with the permission of reference [43]. Copyright © Royal Society of Chemistry 2022.

Ling and co-authors reported a P6A-based theranostic system constructed by means of self-assembly between a water-soluble **P6A3** and a chlorambucil-based azobenzene-containing prodrug (Azo-G) and combination with glucose oxidase (GOx) to form nanoparticles GOx@NPs for the aim of hypoxic tumor diagnosis and controlled therapy [43] (Figure 10).

According to the authors, under hypoxia microenvironment of the tumor cells, GOx@NPs released both the free chlorambucil to provide inhibition of tumor growth and free red fluorescence dye for the tumor imaging (Figure 10). Thus, nanoparticles, GOx@NPs, were nontoxic with respect to HL-7702 cells (normal cells) in vitro, while they expressed remarkable proliferation inhibition abilities against HT-29 cancer cells. In addition, GOx@NPs provided excellent fluorescence diagnosis capability toward hypoxic tumor cells in vivo though selective activation in tumor microenvironment.

## 6. ATP-Triggered Drug Release Pillararene-Based Systems

**Figure 11:**
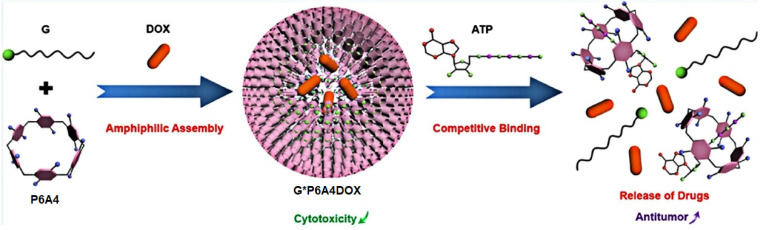
Schematic illustration of the fabrication of a “G*P6A4*DOX” drug delivery system. Reproduced with the permission of reference [87]. Copyright © American Chemical Society 2021.

Li, Meng, and co-authors reported an ATP-triggered P6A-based drug delivery system for smart cancer therapy [87] (Figure 11). To do that, water-soluble pillar[6]arene (**P6A4**) was assembled with sodium decanesulfonate guest (**G**) to form a host–guest assembly, which was confirmed by ^1^H NMR. Upon addition of adenosine triphosphate (ATP), the competitive release of **G** from “G*P6A4” and ATP encapsulation was observed with an association value of 5.67 ± 0.31 × 10^5^ M^−1^. In aqueous solution, the formation of vesicle-structured aggregates was confirmed based on TEM and DLS, with a diameter of ∼100 nm (TEM) to 122.4 nM (DLS). Upon addition of DOX, the morphology and diameter of vesicles changed: based on TEM data, ∼150 nm nanoparticles with dark interiors were formed, and, based on DLS results, their average diameter was 164.2 nm. Within tumor cells, the ATP level is increased to 1–10 mM [88], vs. 1–10 nM in normal tissues [89,90]. To study DOX release, 10 nM and 10 mM ATP concentrations were used, and, at 10 nM ATP, the accumulated DOX release of less than 10% within 16 h vs. 85.1% accumulated DOX release at 10 mM ATP was observed, which was accompanied by an enhancement of the fluorescence of the solution due to free DOX presence. It is worth mentioning that, in in vitro experiments, in the absence of ATP, the “G*P6A4”, even at high concentration, did not exhibit any cytotoxicity towards HepG-2 and LO2 cells.

## 7. *pH*-Controlled Pillararene-Based Systems

**Figure 12:**
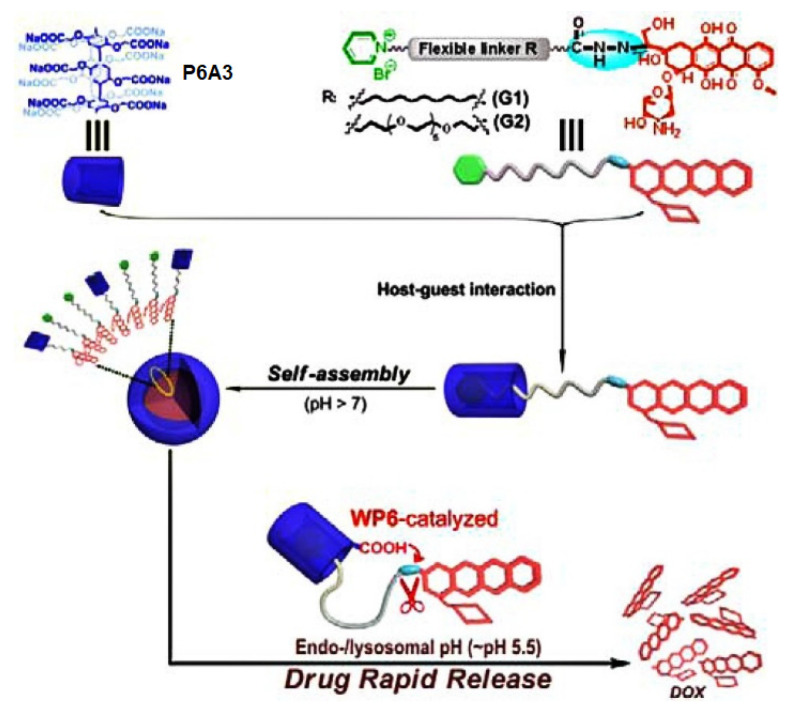
Schematic representation of the construction of the *pH*-controlled drug delivery system based on P6As. Reproduced with the permission of reference [91]. Copyright © American Chemical Society 2015.

Wang, Hu, and coauthors reported P6A-based assembly for controllable *pH*-controlled drug release [91] (Figure 12). To do that, the DOX molecule was conjugated via an acid-cleavable hydrazone bond with a flexible alkyl chain (**G1**) or a short ethylene glycol (**G2**) containing a pyridinium termini. In a weak alkaline phosphate buffer solution, **P6A3** formed amphiphilic host–guest complexes with **G1** or **G2**, which were confirmed by means of ^1^H NMR and fluorescence studies. According to the Tyndall effect and DLS and TEM data, both complexes formed nanoparticles of spherical morphology with an average diameter of 278 and 366 nM, respectively, and with good stability. An extremely high DOX loading content (70 wt %(**G2**) and 85 wt % (**G1**)) and good stability under physiological and weakly alkaline conditions were observed. Both types of nanoparticles exhibited good stability at *pH* 8.0, with the cumulative DOX release of less than 3% within 6 h, while, under simulated physiological conditions (PBS, *pH* 7.4), the cumulative release of DOX was less than 12% within 6 h. Under the simulated endolysosomal environment (*pH* 5.5), a very quick DOX release (100% within 30 min) from “P6A3*G1” and “P6A3*G2” was observed as a result of acid-promoted hydrolysis of hydrazone bonds. According to the authors, at low “P6A3*G1” and “P6A3*G2” concentration (a drug equivalent dosage of 0.1 μg·mL^−1^), the cell viability was more than 80% after 32 h, while, at higher concentration (DOX equivalent dosage of 10.0 μg·mL^−1^), the cell viability decreased significantly. Finally, according to intracellular localization experiments, both types of nanoparticles were taken up by SKOV3 cancer cells (smaller “DOX*P6A3*G1” nanoparticles exhibited faster internalization) via endocytosis, followed by endolysosomal escape and subsequent released DOX distribution in the cytosol and nucleus. It was said that these nanoparticles could efficiently inhibit the proliferation of cancer cells and exhibit potent antitumor activity.

**Figure 13:**
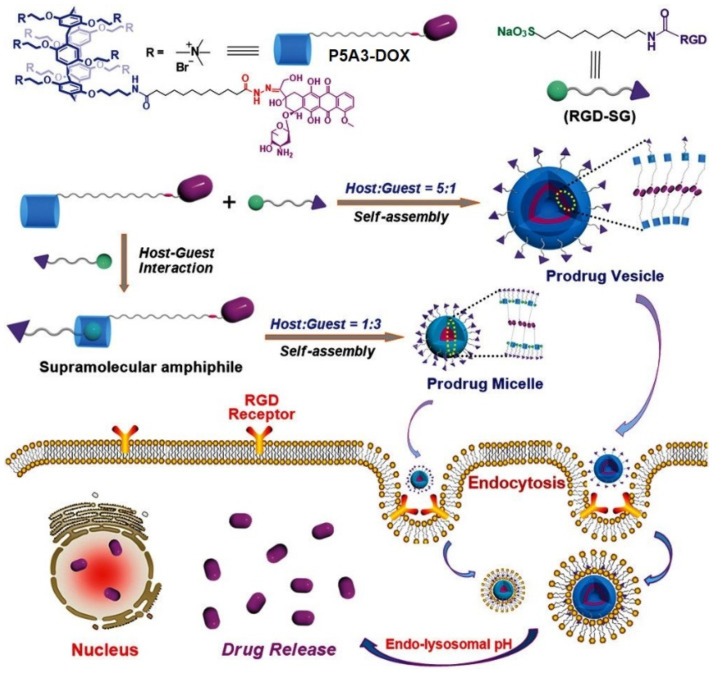
Chemical structures and schematic illustration of the controllable construction of **P5A3**-based tumor-targeting supramolecular prodrug vesicles and micelles for tumor-targeting drug delivery. Reproduced with the permission of reference [92]. Copyright © Wiley 2018.

Hu, Schmuck et al. reported DOX-decorated tumor-targeting nanocarriers based on host–guest recognition between a novel pillar[5]arene-based prodrug “**P5A3-DOX**” with an acid-cleavable linker and an Arg-Gly-Asp (RGD)-modified sulfonate guest RGD-SG [92] (Figure 13). In phosphate-buffered saline, “**P5A3-DOX**” self-assembled into nanoparticles, which exhibited a notable Tyndall effect along with dramatic quenching of DOX fluorescence. In the presence of RGD-SG, the authors observed a fluorescence enhancement due to the formation of two different types of aggregates, with a “**P5A3-DOX/RGD-SG**” ratio of either 5:1 or 1:3. Based on TEM and DLS data, at the host to guest ratio of 5:1, large vesicular structures with diameters around 190 (DLS) or 160 to 200 (TEM) nm were observed, while, at the ratio of 1:3, smaller aggregates with diameters around 10 (DLS) or 4 to 8 (TEM) nm were formed. According to molecular dynamics simulations, the volume ratio of the hydrophilic portion to the volume ratio between the hydrophilic and hydrophobic portions of “**P5A3-DOX**” is responsible for the aggregate formation. It was further demonstrated that the hydrazone linkage in “**P5A3-DOX**” is cleavable under the acidic condition, such as extracellular (*pH* 6.5) and endolysosomal environments (*pH* 5.0) of cancer cells. According to the authors, both the vesicles and micelles were stable under physiological conditions (*pH* 7.4), while DOX release was observed at lower *pH*: 40% DOX was released within 10 h at *pH* 6.5, and, at *pH* 5.0, the cumulative release of DOX was almost 100%. Finally, in in vitro and in vivo experiments, the higher cytotoxicity and more efficient tumor-targeting effect of prodrug micelles on HepG2 and HeLa cancer cells compared to the prodrug vesicles was demonstrated.

**Figure 14:**
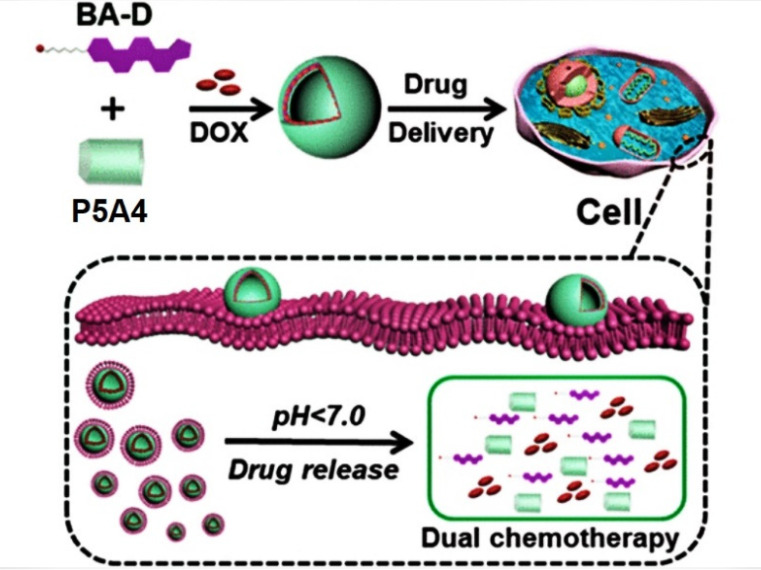
Schematic representation of the *pH*-responsive **P5A4**-based tandem drug delivery system. Reproduced with the permission of reference [93]. Copyright © American Chemical Society 2022.

Very recently, Hu et al. reported a *pH*-responsive P5A-based tandem drug delivery system [93] (Figure 14). Thus, ester bond-linked betulinic acid (BA)-based quaternary ammonium salt amphiphile (BA-D) was combined with **P5A4** to form vesicles, which were loaded with DOX. BA is known to possess a good cytotoxicity toward various type of cancers, while it has a good biocompatibility toward normal cells. The authors claimed that the DOX-loaded “P5A4*BA-D” assembly in acidic media (*pH* = 5.0) efficiently disassembles and realizes both DOX and BA-D, which was observed by using TEM and DLS data. In in vivo experiments with MCF-7 and HepG2 cancer cells, it was confirmed that the loading with DOX of “P5A4*BA-D” assembly enhances its antitumor efficacy due to the synergistic effect of DOX and BA-D with the IC_50_ value for the HepG2 cancer cells of 0.5 µM DOX * BA-D and 0.51 µM DOX-loaded “P5A4*BA-D” vesicles vs. 8.11 µM (BA) and 0.87 µM (DOX) and for the MCF-7 of 0.58 µM DOX* BA-D and 0.61 µM DOX-loaded “P5A*BA-D” vesicles vs. 8.69 µM (BA) and 0.99 µM (DOX). In in vivo experiments with HepG2 tumor-bearing mice, DOX-loaded “P5A4*BA-D” vesicles demonstrated a remarkably reduced systematic toxicity.

**Figure 15:**
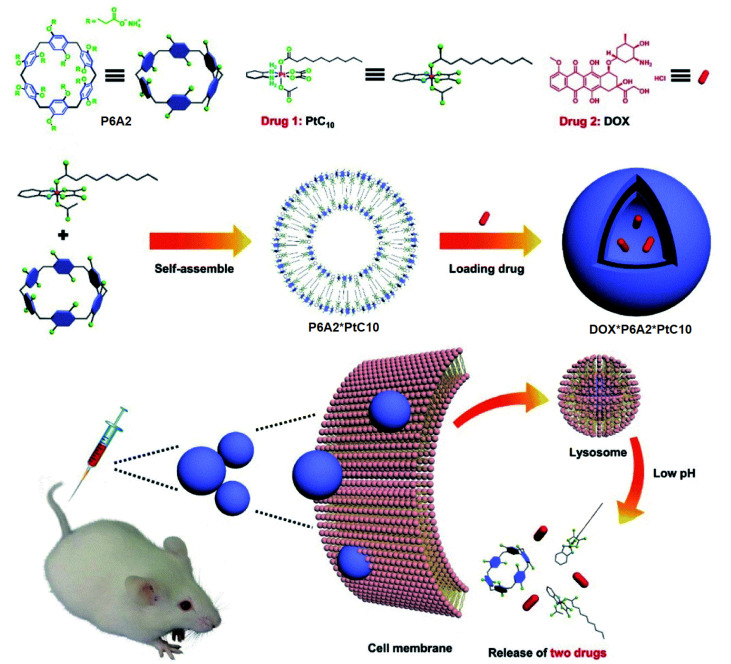
Schematic representation of the *pH*-responsive **P6A2**-based dual drug delivery system. Reproduced with the permission of reference [94]. Copyright © Royal Society Chemistry 2020.

Li, Meng, Sessler, and co-authors reported a *pH*-responsive tandem drug delivery system combined with water-soluble **P6A2**, oxaliplatin (OX)-type Pt(IV) prodrug (PtC10), and DOX [94] (Figure 15). Based on fluorescence titration experiments at *pH* 7.4, **P6A2** readily binds PtC_10_ with a Ka value of (1.2 ± 0.03) × 10^4^ M^−1^, while, at *pH* 5.0, a lower Ka of 1.73 ± 0.15 × 10^3^ M^−1^ was observed, which confirms the *pH* sensitivity of “P6A2* PtC_10_”. It was observed that the *pH* values 7.4 and 5.0 reflect those of normal physiological environment and lysosomes. In aqueous solutions, “P6A2* PtC_10_” forms aggregates in a 1:2 ratio, which was confirmed by fluorescence studies by a notable Tyndall effect. Based on TEM and DLS, the formation of hollow supramolecular vesicles with diameters ranging from 50 nm to 90 nm (TEM) or 91.3 nm (DLS) with the outer wall thickness of 6 nm (TEM) was observed. The obtained hollow vesicles were able to accommodate DOX to form nanoparticles of 100 nm (TEM) or 122 nm (DLS) diameter with dark interiors (TEM), which were stable up to 3 days in water. The acid-triggered DOX-release behavior of “DOX* P6A2* PtC_10_” was then investigated. It was observed that, at *pH* 7.4, approximately 7% of the bound DOX was released from “DOX* P6A2 * PtC_10_” over the course of 24 h, while about 80% within 24 h was seen at *pH* 5.0. For PtC_10_, about 6% was released at *pH* 7.4 and around 70% was released at *pH* 5.0. It was also found that “DOX* P6A2* PtC_10_” could enter *HepG-2* cells via endocytosis to result in intercellular DOX release in the cell nucleus. According to the authors, using “DOX* P6A2 * PtC_10_” provides a synergistic cell cytotoxicity against HepG-2 cancer cells, which is comparable to either PtC_10_ or DOX. Finally, in in vivo tests, the “DOX* P6A2 * PtC_10_” exhibited higher therapeutic efficiency and engendered less body weight loss in HepG-2 tumor xenografts bearing nude mice as compared to various controls.

## 8. Spermine-Driven Drug Release Pillararene-Based Systems

**Figure 16:**
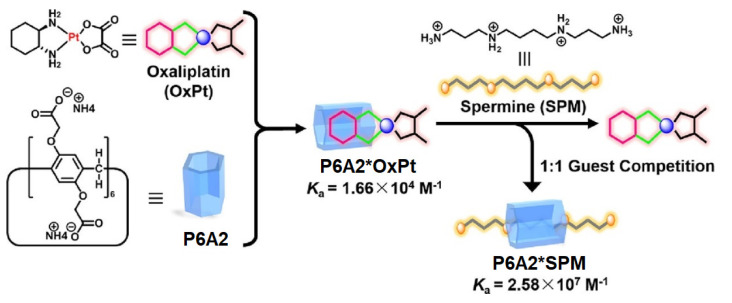
Schematic representation of the spermine-responsive **P6A2**-based OxPt delivery system. Reproduced with the permission of reference [95]. Copyright © American Chemical Society 2018.

As it was mentioned above, in certain types of cancers (lung, colorectal, etc.), the biological amines, such as spermine, are overexpressed. In addition, spermine accommodates readily into the cavity of PAs. Keeping both of these facts in mind, Sun, Zhang, and co-authors reported an assembly between anionic pillar[6]arene host and oxaliplatin (OxPt) as a possible candidate for colorectal cancer chemotherapy [95] (Figure 16).

The authors claimed that the encapsulation of OxPt into the cavity of **P6A2** would reduce its cytotoxicity to the normal cells along with more efficient drug release in the tumor cell microenvironment. At physiological *pH* 7.4, OxPt forms a 1:1 inclusion complex with **P6A2**, which was confirmed based on ^1^H NMR data and Job-plot titration experiments, and, according to the isothermal titration calorimetry (ITC) experiments, the calculated association constant was 1.66 × 10^4^ M^−1^. By using the same approaches, the 1:1 inclusion complex “SPM*P6A2” was confirmed with an association constant of 2.58 × 10^7^ M^−1^. Owing to high cytotoxicity, free OxPt to normal colorectal cells, and low or no cytotoxicity of P6A, the 1:2 “P6A*OxPt” complex was selected for further studies. Moreover, a three-orders-of-magnitude higher binding constant for the “P6A2*SPM” complex compared with the “P6A2*OxPt” one and the competitive binding of SPM by **P6A2** with a simultaneous release of OxPt was expected. According to ^1^H NMR data, the addition of 1.0 equiv SPM to “P6A2*OxPt” results in the release of free OxPt and the formation of a “P6A2*SPM” complex. In in vitro tests with SPM-overexpressed colorectal cancer HCT116 cells, “P6A2*OxPt” exhibited higher anticancer bioactivity than OxPt itself, with an increased amount of about 20% with calculated IC_50_ values for OxPt and “P6A2*OxPt” of about 23.3 and 13.7 μM, respectively, which suggest a notably improved cytotoxicity of “P6A2*OxPt” against colorectal cancer HCT116 in vitro.

## 9. Redox-Responsive Drug Release Pillararene Systems

**Figure 17:**
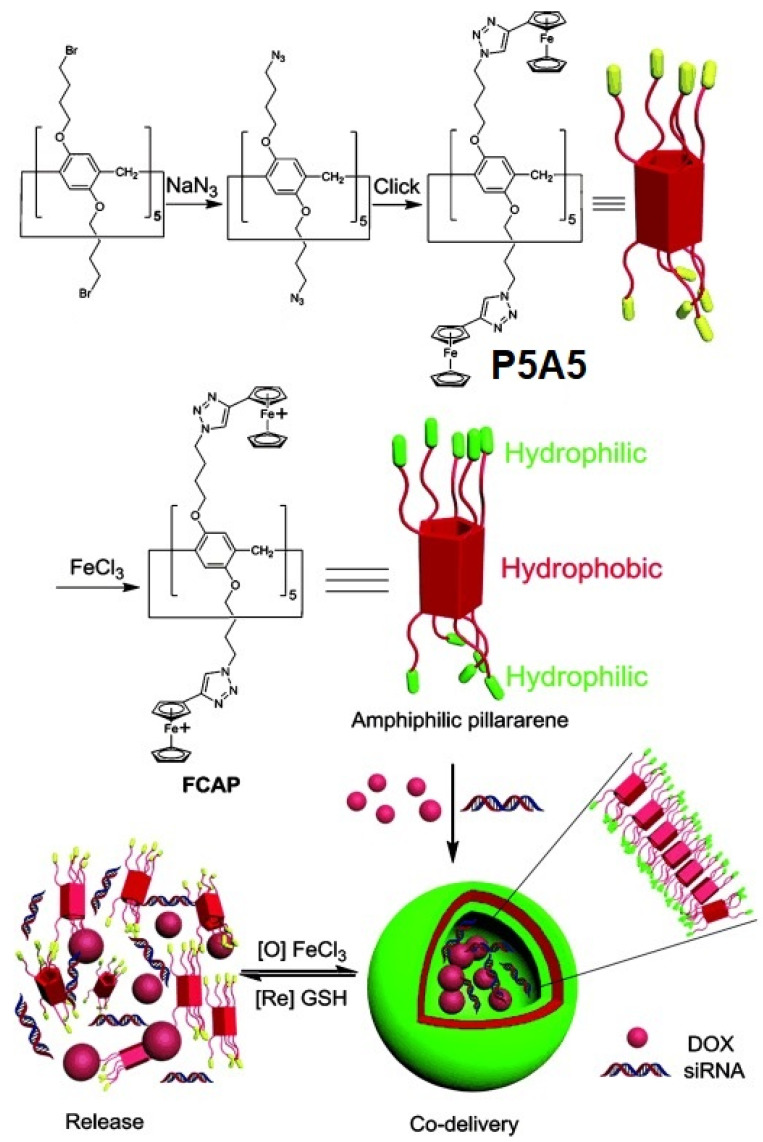
Illustration of the synthesis of **FCAP**, formation of cationic vesicles, and their redox-responsive drug/siRNA release. Reproduced with the permission of reference [17]. Copyright © Wiley 2015.

Y. Pei, Z. Pei, and co-authors reported ferrocenium-capped amphiphilic supramolecular assembly, which is ferrocenium-capped amphiphilic pillar[5]arene (**FCAP**) based on pillar[5]arene **P5A5** for the simultaneous delivery of DOX as cytotoxic drug component and siRNA (MRP1 siRNA) to restore drug sensitivity of cells for more effective chemotherapy via knocking down of drug resistance genes [17] (Figure 17).

**FCAP** were prepared via azide-alkyne cycloaddition (CuAAC) of azide-modified P5A. The following oxidation of the ferrocenyl groups with FeCl_3_ resulted in the formation of amphiphilic **FCAP**, which were able to form regular aggregates in water. According to the SEM, TEM, and DLS data, as well as the observed Tyndall effect, **FCAP** formed stable (in water solution at 300 K for one week) spherical vesicles with the average diameter of 91.9 nm and polydispersity index of 0.298. Thus, the obtained **FCAP** vesicles demonstrated a strong response to GSH (as reductant), which was accompanied by a disappearance of the Tyndall effect. Based on an MTT cell-survival assay, the relative cell viability of 293T cells (normal cells) incubated with unloaded **FCAP** vesicles in the concentrations of 10 μM after 24 h was over 85%, which confirms low toxicity to **FCAP** of normal cells. Next, the experiment on loading **FCAP** with DOX was carried out, and, based on SEM and DLS data, the formation of DOX-loaded vesicles of 79 nm and with a polydispersity index of 0.232 was observed, with DOX encapsulation and loading efficiency calculated to be 67.0% and 9.1% (according to the data of UV/Vis-spectroscopy). Along with good drug loading, the **FCAP** cationic vesicles exhibited GSH-triggering (10 mM concentration of GSH was used, which is relevant to the GSH concentration in cancer cells (1–11 mM) [96]), and DOX release was observed with the amount of DOX up to 92% along with disassembly of DOX-loaded vesicles, while, without GSH exposure, only 39% of DOX was released in the same period. In addition, based the results of in vitro anticancer activity studies using 293T cells and HeLa cells, as well SKOV-3 and KM-12 cells, it was suggested that there is higher cytotoxicity of DOX-loaded **FCAP** against cancer cells, while its cytotoxicity to normal cells was effectively reduced. Finally, siRNA (MRP1 siRNA) was used to fabricate DOX-loaded MRP1 siRNA/cationic vesicle complexes for the co-delivery study to assess the drug resistance gene silencing of SKOV-3 cells. According to the authors, in the case of negative siRNA, used as the control, the cell death and apoptosis of SKOV-3 cells after being incubated with DOX-loaded negative siRNA/cationic vesicle complexes was 37.9%, vs. 52.8% for DOX-loaded MRP1 siRNA/cationic vesicle complexes with an IC_50_ value in SKOV-3 of 4.9 μM vs. 2.1 μM.

## 10. Light-Triggered Drug Release Pillararene Systems

The main challenge for drug delivery systems is their ability to target the tumor and selectively track the process of translocation, drug release, and excretion of the cytotoxic drug. The easiest approach to this involves using fluorescence as an analytic signal and UV/vis irradiation (light) to initiate the drug-release process. Unfortunately, most of the commonly used anticancer drugs are non- or low fluorescent [97,98,99] and cannot be used as fluorescent reporters. The most common approach to solve this issue involves the use of theranostic tools, such as photoactivated drugs [97,98,99], in which the prodrug molecule contains cytotoxic drug combined with a fluorescent reporter molecule by means of a photocleavable linker.

**Figure 18:**
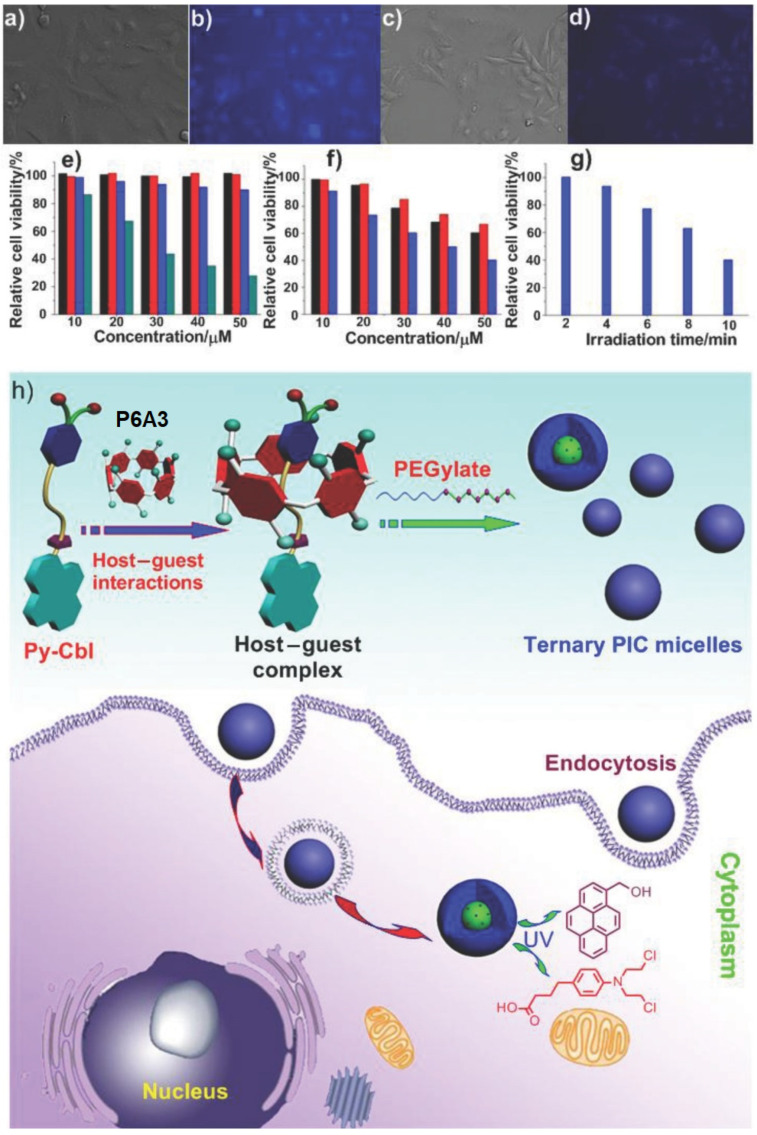
Real-time drug release studies using fluorescence microscopy: (**a**) bright field image of A549 cells incubated with **Py-Cbl** before UV light irradiation; (**b**) fluorescence image of (**a**); (**c**) bright field image of A549 cells incubated with Py-Cbl after UV light irradiation for 10 min; (**d**) fluorescence image of (**c**); (**e**) cell viability tests of Py-Cbl (**black column**), “P6A3*Py-Cbl” (**red column**), ternary complex (**blue column**), and chlorambucil (cyancolumn) against A549 cells; (**f**) cell viability tests of A549 cell line in the presence of different concentrations of **Py-Cbl** (black column), “P6A3*Py-Cbl” (**red column**), and ternary complex (**blue column**) after UV irradiation for 10 min; (**g**) cell viability tests of A549 cell line in the presence of the ternary complex after UV irradiation for different times; the concentration of **Py-Cbl** was 5 × 10^−5^ M; (**h**) schematic illustration of the preparation of ternary PIC micelles and possible cellular pathways; the ternary PIC micelles are endocytosed by cancer cells and chlorambucil is released upon UV irradiation. Reproduced with the permission of reference [100]. Copyright © Wiley 2015.

Following this strategy, Huang and co-authors reported a ternary DDS consisting of photodegradable prodrug (Py-Cbl), in which anticancer drug chlorambucil was combined with pyrene as fluorophore encapsulated into an anionic water-soluble P6A [100] (Figure 18). To enhance membrane permeability, a cationic hydrophilic diblock copolymer methoxy-poly(ethylene glycol)_114_-*block*-poly(_L_-lysine hydrochloride)_200_(PEG-b -PLKC) was introduced into the system of the “P6A3*Py-Cbl” host–guest inclusion complex. The formation of “P6A3*Py-Cbl” with the benzene ring of chlorambucil that penetrated into the P6A cavity was suggested based on DFT calculation of the energy-minimized structure of the host–guest complex. Based on DLS and TEM data, the spherical nanostructures (which were quite stable in buffer for several weeks) from PEG-*b*-PLKC and “P6A3*Py-Cbl” with anaverage size of 80 to 160 nm (TEM) or 127 nm (DLS) were observed. Sharp color contrast between the periphery and central parts of these aggregates suggests their micellar structure. The photo-triggered release of free chlorambucil was confirmed based on the fluorescence studies and the ^1^H NMR experiments. To study the effectiveness of the P6A-based assembly, the A549 cell imaging studies were carried out, and, upon irradiation of UV light after the 24 h incubation of A549 cells with “P6A3*Py-Cbl”, notable changes in spectroscopic behavior and color were monitored due to the photodegradation of Py-Cbl into PyOH and chlorambucil, which resulted in a color change of the cells. Controllable release of Cbl in vitro was verified by using the MTT assay, and the higher cytotoxicity of micelles compared to “P6A3*Py-Cbl” and Cbl and their highest level of toxicity (about 59.7% in comparison to the control) was confirmed.

**Figure 19:**
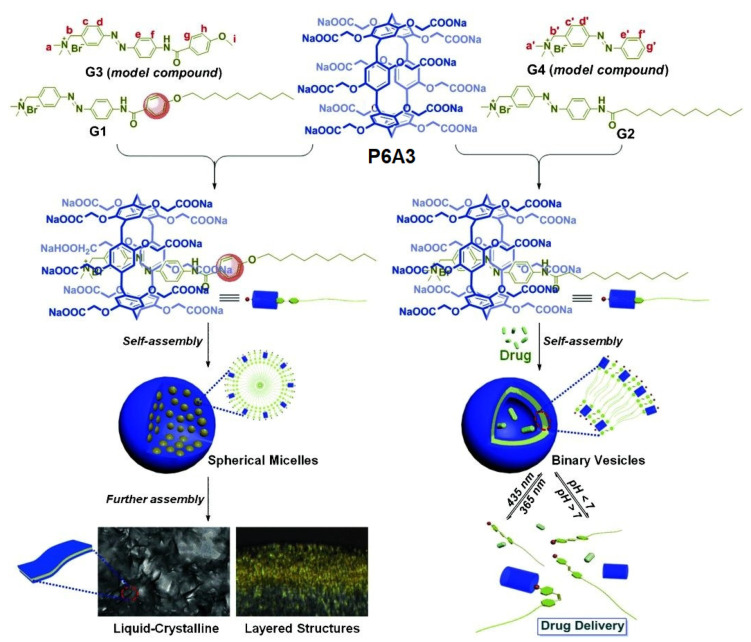
Schematic illustration of the construction of supramolecular micelles “P6A3*G1” or vesicles “P6A3 *G2” and the application of supramolecular vesicles in drug delivery. Reproduced with the permission of reference [101]. Copyright © Wiley 2015.

Wang and co-authors reported two types of supramolecular assemblies, based on water-soluble pillar[6]arene **P6A3** and azobenzene derivatives **G1** or **G2**. Supramolecular micelles formed by **P6A3** with **G1** were gradually transformed into layered structures with liquid-crystalline properties, while **P6A** and **G2** formed supramolecular vesicles as photo- and *pH*-responsive drug delivery systems [101] (Figure 19).

Due to **G1** and **G2** poor water solubility, to study the host–guest complexation in the D_2_O model, guests **G3** and **G4** were used, and, based on the results of ^1^H NMR, ^1^H–^1^H COSY, and 2D ROESY spectroscopy, the formation of “P6A3*G3” and “P6A3*G4” 1:1 inclusion complexes was observed with association constants of (1.03 ± 0.24)×10^4^ M^−1^ and (2.06 ± 0.88) × 10^4^ M^−1^, respectively. The amphiphilic “P6A3*G1” and “P6A3*G2” supramolecular complexes were further prepared. Based on the observed Tyndall effect and TEM and DLS data, there was formation of “P6A3*G1” aggregates as solid spherical micelles of about 220 nm average diameter (DLS), and, for the “P6A3*G2”**,** the formation of vesicles of hollow spherical morphology with a diameter of 160 nm (DLS), or from 100 to 200 nm (TEM), was confirmed. According to the authors, in “P6A3*G2”, the Tyndall effect disappeared after adjusting the solution *pH* to 6.2 and no vesicles were detected in TEM image, and, after adjusting the *pH* value to 7.4, the re-formation of the vesicle was observed, which was confirmed by DLS data. As for proto-response, according to TEM data, upon irradiation of the “P6A3*G2”with UV light at 365 nm, most of the vesicles collapsed due to the E/Z isomerization (*trans-* to *cis-*) of the azabenzene moiety of **G2,** and only a few irregular aggregates could be observed, while, upon irradiation with visible light at 435 nm, re-formation of the vesicle was observed, which was confirmed by DLS data. After loading of “P6A3*G2”vesicles with Mitoxantrone (MTZ), based on TEM data, the authors detected the MTZ loading into the vesicle cavities with the formation of MTZ-loaded “P6A3*G2”of larger size (≈364 nm), compared to MTZ-free vesicles (≈160 nm), and, based on UV/Vis absorption spectra, the MTZ encapsulation efficiency was calculated to be 76%. The authors claimed that, under simulated physiological conditions (*pH* 7.4), the cumulative leakage of MTZ from MTZ-loaded vesicles was less than 8% within 12 h, while 44% and 93% were detected at *pH* 6.4 and *pH* 4.4 within 12 h with an intensive MTZ release in the first 2 h. In addition, under UV irradiation with UV light, the cumulative amount of released drug was about 60% after 4 h. In in vitro tests with MCF-7 cancer cells, the relative cell viability of cancer cells with UV irradiation was only 20% after 96 h, which showed similar therapeutic effects to the free MTZ (17%), whereas without irradiation was about 27%. In addition, MTZ-loaded “P6A3*G2”vesicles could induce apoptosis in MCF-7 cells and, after 48 h, the quantity of apoptotic cells of the whole tested MCF-7 cells was about 47.2%, while, after 96 h, the ratio of apoptotic cells significantly increased to 70.2% and the majority of apoptotic cells were the early apoptotic cells.

## 11. Multi-Response Drug Release Pillararene Systems

**Figure 20:**
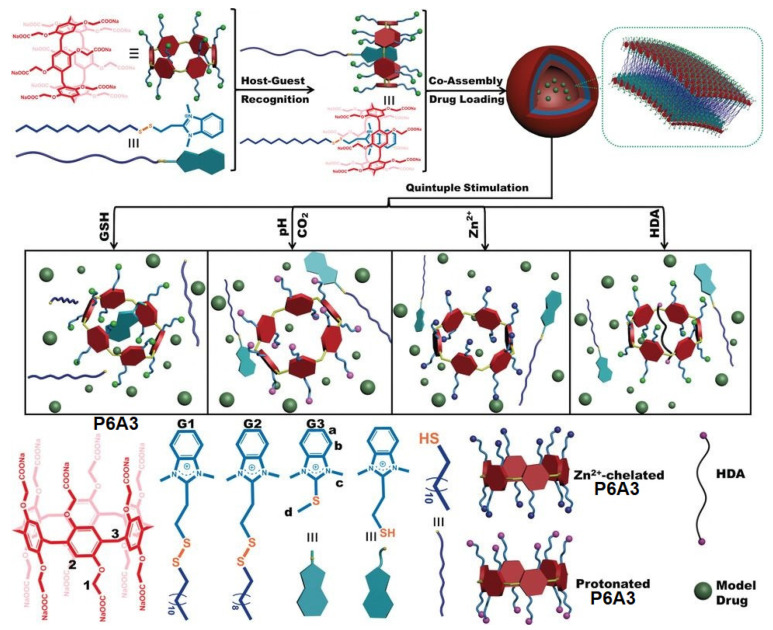
Chemical structures of **P6A3** and **G1–G3** and illustration of their inclusion complex and DOX-loaded vesicles for controlled drug release. Reproduced from reference [102]. Copyright © Wiley 2017.

Du and co-workers reported a quintuple stimulated drug delivery system based on pillar[6]arene **P6A3** [102] (Figure 20). In this system, **P6A3** was assembled with disulfide-linked benzimidazolium amphiphiles **G1–G3** to form amphiphilic vesicles. Up to five stimuli for the encapsulated drug release were reported by the authors, such as glutathione, which could break the S-S bond in the surfactant, *pH* or CO_2_, which could change COO^−^ group to COOH group, Zn^2+^, which was able to chelate with **P6A3**, and, finally, hexanediamine (HDA), which existed in deprotonated form under these conditions and blocked the cavity of **P6A3**.

## 12. Insulin Delivery Systems Based on Pillararenes

**Figure 21:**
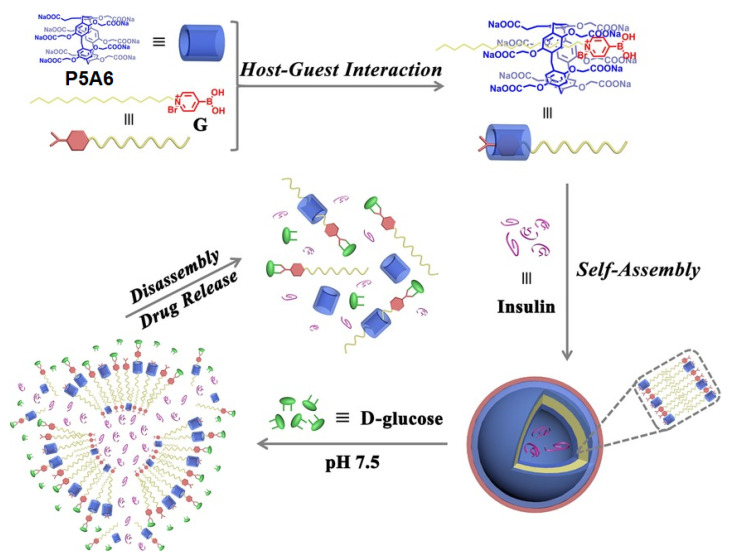
Schematic illustration of the formation of supramolecular vesicles based on “P5A6*G” supra-amphiphile and their glucose-responsive insulin release. Reproduced from reference [104]. Copyright © Wiley 2017.

Type 1 diabetes mellitus (T1DM), juvenile diabetes, or insulin-dependent diabetes, is an autoimmune disease that leads to the destruction of insulin-producing pancreatic beta cells, causing an absolute deficiency of insulin. People with T1DM require life-long insulin replacement therapy along with blood glucose level monitoring. The insulin replacement therapy is provided via multiple daily insulin injections, or, more preferably, via continuous subcutaneous insulin infusion by using an insulin pump. In addition, oral insulin is still a main challenge for research and development due to the physiological barriers to its absorption, its low bioavailability, low biopotency, and others [103]. Therefore, the insulin delivery systems combined with real-time glucose monitoring devices are of high demand. As a first example of such kinds of systems, Wang, Hu, and co-authors reported a supramolecular assembly based on water-soluble pillar[5]arene (**P5A6**) and pyridine boronic acid with a pyridinium moiety bearing at N1 atom long aliphatic chains as an amphiphilic part (**G**) [104] (Figure 21). The authors suggested the possibility of the inclusion of the alkyl chain and partial pyridine group of **G** into the hydrophobic cavity **P5A6** and the formation of “P5A6*G” inclusion complex, and that was confirmed by the ^1^H NMR experiment in D_2_O using a modified pyridinium salt (**MG**) with a shorter butyl chain at N1. The ability of “P5A6*G” supra-amphiphile to form higher-order aggregates in the aqueous phase was confirmed based on the Tyndall effect, DLS experiments, and TEM images: the obtained aggregates showed a hollow spherical morphology with a diameter from 120 nm (according to the data of TEM) to 132 nm (DLS). Boronic acids are commonly used for the recognition of carbohydrates at physiological conditions [105,106,107], and, therefore, the boronic acid pyridinium moiety in “P5A6*G” could form a stable boronate ester with the glucose. The response of “P5A6*G” to D-glucose was confirmed by means of ^1^H and ^11^B NMR. In addition, due to the strong bonding between boric acid ligand **G** and D-glucose, the authors observed no “P5A6*G” vesicles in the TEM image and the disappearance of the Tyndall effect. Along with D-glucose binding, the obtained “P5A6*G” demonstrated the ability to accumulate DOX, which is better than in the absence of D-glucose. More importantly, “P5A6*G” vesicles exhibited the ability to encapsulate insulin to form insulin-loaded vesicles, and, according to TEM and DLS results, these vesicles were much larger (ca. 320 nm) compared to unloaded “P5A6*G” (ca. 132 nm) and the vesicles adopted an irregular morphology. In the in vitro insulin-release experiments, the authors observed almost no insulin release under physiological conditions (*pH* 7.4). In addition, in the normal blood glucose level (1.0 mg·mL^−1^), the cumulative release amount of insulin was only about 20% within 60 min, indicating that unwanted insulin release would not occur under normal blood glucose levels, and the cumulative release efficiency increased to ca. 60% at 11.1 mM glucose and ca. 70% at 30.5 mM glucose. In in vitro tests, “P5A6*G” exhibited good cytocompatibility for MRC-5 normal cells. Based on all the above, these vesicles can be used as nanocarriers for insulin delivery.

**Figure 22:**
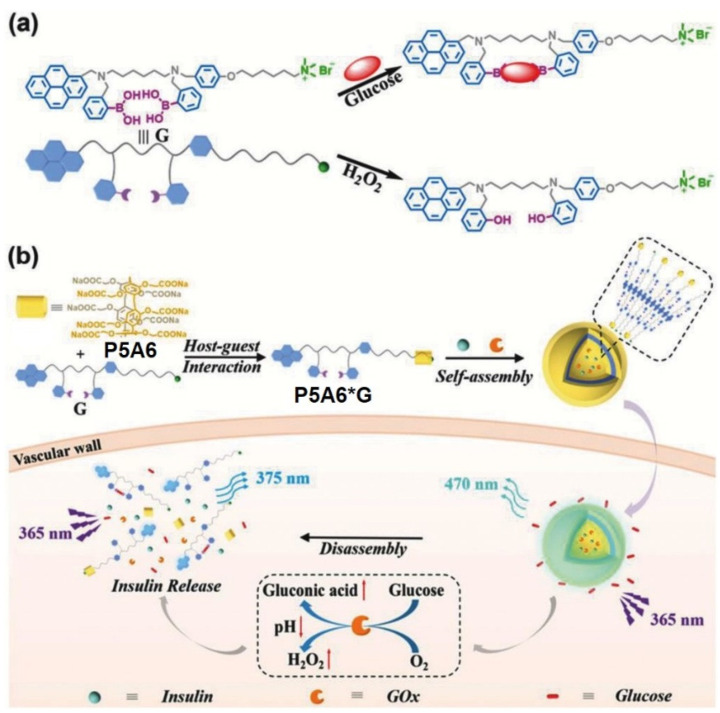
Schematic illustration of the glucose-responsive supramolecular insulin delivery system. (**a**) Chemical structure and the mechanism of the multiresponsive diphenylboronic acid guest **G**. (**b**) Supramolecular self-assembly of the host–guest complex “P5A6*G” into vesicles and their successful encapsulation of insulin and GOx as well as the efficient insulin release under hyperglycemic state. Reproduced from reference [108]. Copyright © Wiley 2018.

The same group recently reported a nanovesicle based on water-soluble pillar[5]arene **P5A6**, pyrene fluorophores connected with a long alkyl-chain quaternary ammonium salt bearing two phenyl boronic acid moieties appended and loaded with insulin and glucose oxidase (GOx) [108] (Figure 22). According to the authors, the obtained insulin-GOx-loaded supramolecular assembly can serve as selective glucose excimeric fluorescence turn-on sensor and a controlled insulin delivery actuator, as insulin and GOx are simultaneously encapsulated in the vesicles. In the presence of glucose, the structure disruption of this supramolecular assembly occurs to result in an efficient encapsulated insulin. It was observed that the entrapped insulin release could be triggered by either high glucose concentration or in situ-generated H_2_O_2_ and acid microenvironment due to the GOx-promoted glucose oxidation to result in gluconic acid. In the experiments on insulin release, the authors observed the slow release of insulin at the glucose concentration of (100 mg·dL^−1^), while, at the concentration of glucose of 250 mg·dL^−1^ mM, the most efficient insulin release (40%) was observed, which confirms the sensitivity of insulin-GOx-loaded vesicles were unsensitive to a normal concentration of glucose and quite sensitive to the condition of hyperglycemia.

**Figure 23:**
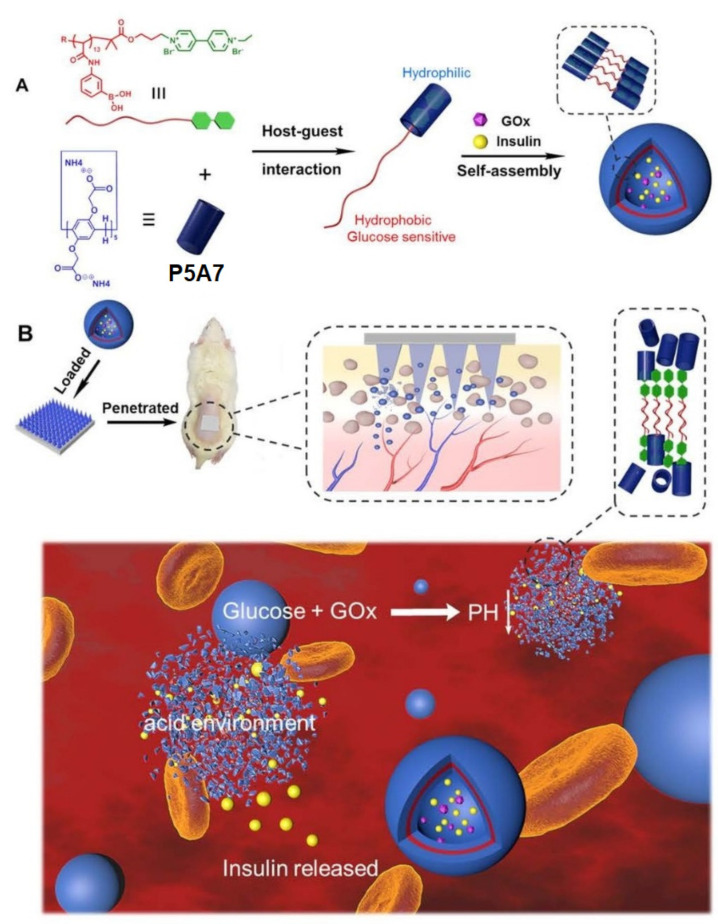
Schematic illustration of a stimuli-responsive P5A-based system and transdermal insulin delivery in a diabetic rat. (**A**): the preparation process of the stimuli-responsive system based on host−guest interaction; (**B**): drug-loaded patch for insulin release in vivo. Reproduced with the permission of reference [109]. Copyright © American Chemical Society 2020.

Another example of a glucose-responsive PA-based insulin delivery/release system was reported by Tong, Cen, and co-authors [109] (Figure 23). To construct this nanocarrier pillar[5]arene, **P5A7** was assembled with paraquat-terminated poly(phenylboronic acid) (**PPBA-G**) with a binding constant of (8.20 ± 1.70) × 10^4^ M^−1^. According to the DLS data in phosphate-buffered saline (PBS) solution (*pH* = 7.4), the formation of supramolecular polymer vesicles (PVs) of 50 nm was detected. It was observed that the presence in these PVs of glucose-responsive PPBA-G and *pH*-responsive P5A results in dual-responsiveness of the obtained PVs. Thus, according to DLS data, at the normal glucose concentration (100 mg·dL^−1^), the PVs did not show any changes, while they totally disappeared at hyperglycemic conditions, namely at the 400 mg·dL^−1^ glucose concentration. On the other hand, by changing solution *pH* levels, the authors observed the disassociation of “P5A7*PPBA-G” host−guest assembly, resulting in solid spheres (**PPBA-G**) at *pH* 5.0 and the reconstruction of the PVs structure at the physiological *pH* of 7.4. After both insulin and GOx are simultaneously encapsulated within the PVs, their insulin-release ability was studied. According to the authors, the obtained insulin-Gox-loaded PVs demonstrated fast insulin release (90%) under the hyperglycemia conditions (400 mg·dL^−1^), as well as at *pH* levels of 5.0 (60%), which confirms their dual-stimuliresponse. In addition, in cell survival tests, the cell viability of 3T3 mouse fibroblast cells cultured with the above-mentioned PVs were all above 90%, implying the low cytotoxicity of all drug-loaded PVs. Finally, the ability of these PVs for transdermal insulin delivery by using PVA/PVP microneedle patches was carried out and the appealability of this platform, probably for the treatment of diabetes in vivo, was suggested.

## 13. Conclusions and Perspectives

In summary, pillararenes represent a promising class of molecular hosts for targeted drug delivery. Due to a possibility for the modification of both ends of the pillararene cavity, neutral, cationic, anionic, or amphiphilic pillararenes can be easily prepared.

Based on X-ray data, the cavity size of pillar[5]arenes is ca. 4.7 Å [1,110], and this is similar to the size of such macrocycles as α-cyclodextrin and cucurbit[5]uril. The cavity size of pillar[6]arenes is larger (ca. 7.5 Å) [111], and this cavity size is similar to that of β-cyclodextrin and cucurbit[6]uril. Pillar[5]arenes are able to form inclusion complexes with linear alkanes, as well as pyridinium and ammonium cations [110], while pillar[6]arenes are able to form inclusion complexes with bulky hydrocarbons [110], as well as with bulky ferrocenium [8], tropylium cations [110], n-octyltriethyl ammonium hexafluoro-phosphate, 1-adamantylammonium tetrakis[3,5-bis(trifluoromethyl)phenyl]borate, diquat, benzimidazolium, methylene Blue, etc. [112]. The formation of the inclusion complexes seems to not influence the pillararene geometry due to its high rigidity.

In some cases, the pillararenes themselves can exhibit a certain biological activity, for instance, antibiofilm activity. For the construction of pillararenes-based targeted drug delivery systems and the following drug delivery mechanism, two main strategies are involved: (1) direct formation of a host–guest inclusion complex with small drug-like, drug, or prodrug molecules and (2) the initial formation of amphiphilic pillarene–guest complexes with their following aggregation to result in vesicles or micelles, which are able to encapsulate drug molecules of various geometry and size. The first approach, however, is very limited by the guest molecule size and/or geometry and the drug loading degree. The mechanism for the encapsulated drug release involves the cleavage of the linker moiety of prodrug under external stimuli, such as chemical reaction or photochemical transformation, or by means of competitive binding with other guest molecules, for instance, compounds presented in pathological microenvironments.

The second approach involves the encapsulation of drug molecules within the pillar-arene-based supramolecular vesicles or micelles and the following disaggregation of these drug-loaded aggregates under external stimuli, such as light, oxidants, or chemicals present in the paralogical processes microenvironment. The advantages of such an approach are high drug loading value, multi-stimuli response, as well as possibility for encapsulation of drug/pre-drug molecules of various nature, geometry, and size. At the same time, these pillararene-based supramolecular delivery systems are silent under normal physiological conditions. At the moment, various pillararenes-based systems for the delivery of different cargoes are reported from antibiotics and anti-cancer drugs to *siRNAs* and insulin.

In many cases, targeting groups are not used in reported pillararene-based drug delivery systems, except the several examples of insulin delivery systems, in which boronic acids moieties are used to target sugars [104,105,106,107,108,109]. In the case of other pillararene-based drug delivery systems, the macrocycle ring substitution is used for improving their performance, and not for targeting. It is worth to mention that Hu, Schmuck et al. [92] reported a pillararene-based DOX nanocarrier with an incorporated Arginylglycylaspartic acid (RGD) targeting moiety, which was recognized and bound by the cell adhesion proteins, integrins. In addition, several small-molecule-based or macrocyclic systems [113,114,115], including a pillarerene-based one [116], bearing mitochondria targeting triphenylphosphonium moieties, were reported as diagnostic and therapeutic tools for various types of cancers. Thus, these moieties may be suggested to be introduced in pillararene-based anticancer drug delivery systems.

As for future perspectives on pillararene-based drug delivery systems, one also can mention the lack of reports on the application of pillararenes as carriers for antiviral drug delivery, while antiviral drugs carriers were reported for other classes of macrocycles [117,118]. Additionally, due to a wide range of binding motifs, pillararenes offer the potential to interact directly with viruses and inhibit infection, for instance, it was reported for human papillomavirus [119]. To date, the use of other macrocycles as antivirals is also limited by few examples [120,121], including a recent report on the inhibition of a large variety of viruses, including herpes simplex virus 2 (HSV-2), respiratory syncytial virus (RSV), and SARS-CoV-2, by cucurbit[n]urils via host–guest supramolecular interactions between viral surface proteins and the cavity of cucurbit[n]urils [122].

## Data Availability

The extra data are available from the authors.
